# Induction of IFNα or IL-12 depends on differentiation of THP-1 cells in dengue infections without and with antibody enhancement

**DOI:** 10.1186/1471-2334-12-340

**Published:** 2012-12-08

**Authors:** Rong-Fu Chen, Lin Wang, Jiin-Tsuey Cheng, Kuender D Yang

**Affiliations:** 1 Department of Medical Research and Development, Show Chwan Health Care System, Changhua, Taiwan; 2 Department of Pediatrics, Chang Gung Memorial Hospital-Kaohsiung Medical Center, Chang Gung University College of Medicine, Kaohsiung, Taiwan; 3 Department of Biological Sciences, National Sun Yat-sen University, Kaohsiung, Taiwan

**Keywords:** Monocyte, Deneritic cells, Differentiation, CD123, IFNα, IL-12

## Abstract

**Background:**

Appropriate induction of the early Th1 cytokine IL-12 is a critical defense directed against viral infection. We have previously shown that different viruses elicited either IL-12 or IFNα dependent Th1 reactions. Using dengue-2 virus, we sought to explore how dengue-2 induced IL-12 or IFNα expression by monocytic and its derived dendritic cells.

**Methods:**

We employed human monocytic cell line, THP-1, to investigate whether differentiation of monocytic cells is involved in the switch between IFNα and IL-12 induction. Flow cytometry, RT-PCR and ELISA were respectively used to determine cell differentiation, IL-12 and IFNα mRNA expression and protein production.

**Results:**

THP-1, expressing CD123, which is a plasmacytoid dendritic cell marker, but not CD14, CD11b or CD11c revealed IFNα mRNA expression while stimulated by dengue-2. In contrast, PMA-induced THP-1 differentiation toward monocytic cells expressed CD11b^+^, and CD14^+^, but not CD123, and revealed exclusively IL-12 expression while stimulated by dengue-2. Further studies showed that CD123^+ ^expressing THP-1 cells elicited higher IFNα expression in dose and time dependent induction after infection, and PMA-induced monocytic differentiation of THP-1 cells revealed IL-12 expression. Antibody-dependent enhancement of DEN-2 infection significantly suppressed the DEN-2 induced IL-12 p40 expression in monocytic differentiated THP-1 cells.

**Conclusions:**

Clarification and modulation of the early Th1 reaction in different monocytic cells may change or prevent complication from dengue infection.

## Background

Dengue fever (DF) is transmitted by mosquitoe bite which introduces dengue viruses (DEN) including 4 serotypes. DF is highly prevalent in tropical and subtropical areas, with at least 50 million new cases each year [1,2]. Occasional, DF progresses to dengue hemorrhagic fever (DHF), a potentially life threatening complication associated with vascular leakage, hemorrhage, thrombocytopenia and shock [3-5]. More than 1 billion people are at risk of dengue infection and there are approximately 100 million cases of DF and 500,000 cases of DHF every year [6]. DHF has been classified into four grades on the basis of clinical presentation. The mildest is grade I and the most severe is grade IV [7,8]. The first indication of an immunological mechanism for DHF was the observation in a Bangkok outbreak of DHF in 1960s [9]. In that outbreak, over 85% of children with DHF had high DEN heterotypic cross-reactive antibody titers, suggesting an antibody-dependent enhancement (ADE) of DEN infection in the pathogenesis [10]. Monocytes, macrophages and dendritic cells (DCs) play a crucial role in immune responses against virus infection, in which IL-12 and IFN α/β are early mediators of Th1 cell immunity [11-13]. It is well known that IL-12 derived from monocytes or dendritic cells can polarize Th1 reaction [14-16]. Whether type I interferons are involved in IFNγ induction mediating Th1 reaction remains controversial [17,18]. Recently, we have found that different microorganisms could induce IL-12 or IFNα to polarize Th1 reaction in a mutually exclusive fashion [16]. Different viruses induce IL-12 or IFNα mutually exclusive is usually mediated via ligation of different toll-like receptors (TLRs) [19-21].

DCs play a central role in the initiation of T cell-mediated antiviral immune responses [22,23]. Different DCs release different cytokines to polarize T helper (Th) cells. Plasmacytoid DCs release IFN α/β to polarize Th1 reaction [24], myeloid DCs release mainly IL-12 to enhance Th1 reaction [25], and regulatory DCs release IL-10 or TGFβ to polarize Treg [26]. Besides, DCs are very effective at taking up antigen, by pinocytosis of soluble compounds, macropinocytosis of high-molecular-weight antigens and phagocytosis of particles [27]. After encountering antigen, DCs migrate to the lymph nodes where DCs activate specific T cells [28]. The migration is dependent on the expression of chemokine receptors, which enable the cells to sense chemotactic gradients, and cell adhesion molecules for interaction with endothelial cells. Several of these molecules play important regulatory roles, such as ALCAM, which is expressed on activated T cells and on monocyte-derived DCs, and might play a role in DC migration [29].

We have previously shown that DHF, a potential fatal complication of DF, is associated with higher Th2 reaction [30-32] and lower Th1 polarization [33]. Moreover, we have also demonstrated that varicella-zoster virus and Bacillus Calmette-Guerin (BCG) induce IFNα and IL-12 production, respectively [16]. This study was conducted to investigate whether differentiation of monocytic cells is involved in the switch between IFNα and IL-12 induction after dengue infection, and whether ADE of dengue infection could modulate the production of IFNα and IL-12 by different monocytic cells.

Monocytes and macrophages are recruited to sites of inflammation and play critical roles in innate host defense mechanisms. THP-1, human promonocytic leukemia cells, grow as nonadherent promonocytes, and differentiate to macrophage-like cells upon treatment with PMA [34,35]. PMA, a potent activator of PKC, arrests THP-1 proliferation and induces the expression of several macrophage characteristics [36-38]. It remains unclear whether THP-1 monocytic cells can express IFNα or IL-12, depending on differentiation; or THP-1 monocytic can develop into different DCs, responsible for exclusive IFNα or IL-12 expression. ADE of dengue has long been implicated in severe, usually secondary, DEN infections. Preexisting heterotypic antibodies of dengue might promote dengue infection via their Fc-gamma receptor (FcγR) [39], this may not only facilitate the virus’ entry, but also modifies innate and adaptive immune responses [40]. Employing DEN-2 virus, which is known to cause the most outbreaks of DHF, associated with altered Th1/Th2 reaction [41-45], we attempted to explore DEN-2 virus induction of IFNα or IL-12 in a monocytic differentiation model.

## Methods

### Reagents and medium

Phorbol 12-myristate 13-acetate (PMA), and dimethyl sulfoxide (DMSO) were purchased from Sigma Chemical Co. (St. Louis, MO). RPMI 1640 culture medium was obtained from Gibco BRL (Grand Island, NY, USA). Fetal bovine serum (FBS), gentamicin, and L-glutamine were purchased from Gibco BRL, Inc. (Grand Island, N.Y., USA).

### Cells culture

Monocytic THP-1 cells were grown in RPMI 1640, 10% FBS (endotoxin level <0.25 EU/mL; Gibco BRL, Grand Island, N.Y., USA), 2 m*M* L-glutamine (Gibco BRL, Grand Island, N.Y., USA) at 37°C, and 5% CO_2_ incubator. THP-1 cells (2×10^5 ^cells/ml) were subcultured every 3 days, and PMA (8 nM) was used to induce THP-1 cell differentiation. To study time dependent effect, cells (2×10^6 ^cells/ml) were used for studies with dengue-2 infection at multiplicity of infection (MOI) = 1.0 for 6 to 72 hours as indicated. For studying different infection dose, we used MOI from 0.1, 0.5, 1, 5 and 10 to study its dose-dependent effect.

### Dengue-2 virus preparation

Dengue virus type 2 (DEN-2, New Guinea C strain, ATCC) was obtained from the Institute of Preventive Medicine, National Defense Medical Center, Taipei. Viruses were propagated in C6/36 mosquito cells in Eagle's minimal essential medium (MEM) (Gibco BRL, Grand Island, N.Y., USA) containing nonessential amino acids (Gibco BRL, Grand Island, N.Y., USA), 1% sodium pyruvate, 0.2% sodium bicarbonate and supplemented with 1% antibiotic (Gibco BRL, Grand Island, N.Y., USA) and 10% heat-inactivated FBS at 28°C for 5 days. Baby hamster kidney cells (BHK-21) were grown in MEM as described above. A large collection of virus culture was pooled and showed a titer of 1.0 × 10^7^ PFU/ml determined by real-time quantitative RT-PCR as previously described [30]. All experiments about DEN infection were set at MOI = 1.

### Flow cytometric analyses of cell surface markers

In order to characterize the change of cell differentiation markers on THP-1 cells, we measured pDC-specific and mDC-specific markers on THP-1 cells by flow cytometry. These cells (2 × 10^6^ cells/ml) are treated with and without 8 nM PMA for 72 hours. Cell surface stainings were performed by direct immunofluorescent assay with fluorescence-conjugated mAbs (CD14-PE, CD11b-PE, CD11c-PE, and CD123-FITC) and corresponding isotype control antibodies for 30 minutes. After washing in PBS twice, cells were fixed in 2% paraformaldehyde for 20 min, washed, and resuspended at ~10^6^ cells per milliliter before acquisition.

### Real-time quantitative RT-PCR analysis of IL12B and IFNα mRNA expression

We subjected total RNA extracted from THP-1 cells with and without differentiation treatment to quantitative analysis of mRNA expression of IL12B and IFNα. In brief, the cell pellet was mixed with 0.5 ml of Tri-Zol solution (Invitrogen, California, USA). After thorough vortexing, samples were added 0.1 ml of chloroform (Scharlau, sa, Barcelona, European Union) for phase separation. After centrifugation, the upper aqueous phase was transferred to a fresh DEPC-treated eppendorf and the same volume of isopropanol (Merck KGaA, Darmstadt, Germany) was added for RNA precipitation at −20°C for 1 hour. The RNA was harvested by centrifugation at 12,000 × g for 10 minutes at 4°C, followed by 75% ethanol (Merck KGaA, Darmstadt, Germany) precipitation. Finally, the RNA was subjected to the real-time RT-PCR detection with SYBR Green PCR reagents (RealQ-PCR Master Mix Kit, Ampliqon) using the ABI PRISM 7500 instrument (Applied Biosystems, Foster City, CA) as previously described [46]. Primers for the quantitative detection of target mRNAs were designed by using Primer Express computer software (Applied Biosystem, Foster City, CA). For the IL12B gene, the primer sequences were 5’- acctccacctgccgagaat-3’ (forward) and 5’- acctccacctgccgagaat-3’ (reverse). For the IFNα. gene, the primer sequences were 5’-atttctgctctgacaacctc-3’ (forward) and 5’-tgacagagactcccctgatg-3’ (reverse) [47]. In order to evaluate PCR efficiency, a 3 replicates and 5-log dilution series were performed. A slope of −3.3 ±10% reflects an efficiency of 100% ±10%. Samples were analyzed in three independent duplicate experiments. The RT-PCR cycling parameters were set as follows: the RT reaction at 50°C, 2 minutes; 60°C, 30 minutes; and 95°C, 5 minutes; followed by 40 cycles of PCR reactions at 94°C, 20 seconds, and 60°C, 1 minute. The results were detected in a real-time fashion and recorded on a plot showing fluorescence vs. time. RT-PCR products were also visualized on ethidium bromide-stained 1.5% agarose (Pierce Co., Rockford, IL, USA) gel with a 100-bp ladder (Pharmacia Biotech, Piscataway, NJ, USA) as a reference. The increase of the IL12B and IFNα mRNA expression were therefore calculated assuming 100% efficient PCR where each C_t_ was normalized to β-actin mRNA expression as shown by the equation at 2 ^[Ct1(target) − Ct1(actin)][Ct2(target) − Ct2(actin)]^. The C_t_1 (target) and C_t_2 (target) represent the C_t_ values for the IL12B or IFNα gene expression in treated and mock samples, respectively. C_t_1 (actin) and C_t_2 (actin) represent the C_t_ values for the β-actin gene.

#### Measurement of IFNa, and IL-12 p40 levels

To measure IL-12 protein production, 10^6^ THP-1 cells with or without PMA treatment per well are seeded into 1-ml culture medium in triplicates in 24-well tissue-culture plates and incubated at 37°C in humidified 5% CO_2_ atmosphere in the presence or absence of different stimuli, as indicated above. After 24 and 72 hours, cell-free culture supernatants are removed and assayed for multi-species IFN-α (PBL Biomedical Laboratories, Endogene Inc., Cambridge, MA, USA), IL-12 p40 (R&D Systems, Minneapolis, MN, USA) concentrations using ELISA. The results were calculated from interpolation in a standard curve made from a series of well-known concentrations of commercial standards [30,32].

### Assessment of antibody-dependent enhancement of virus infections in differentiated THP-1 cells

To assess ADE, DEN-2 viruses were preopsonized with and without DEN-1 or DEN-2 immune sera at 4°C for 60 min as previously described [32], PMA-differentiated THP-1 cells (2 x 10^5^cells/ml) were incubated with DEN-2 virus with and without DEN immune sera at MOI of 1 at 37°C for 60 min. The infected cells were washed to remove extracellular viruses before culture in RPMI 1640. The infected PMA-differentiated THP-1 cells were cultured for 24 hours before the culture supernatants were harvested for determination of DEN-2 virus titers by real-time RT-PCR assay for 40 cycles using TaqMan technology (Applied Biosystems, Foster City, CA). The forward primer, reverse primer and nested fluorescent probe sequence for detecting DEN-2 were 5'-GGC TTA GCG CTC ACA TCC A-3', 5'-GCT GGC CAC CCT CTC TTC TT-3', and FAM-CGC CCA CCA CTA TAG CTG CCG GA-TAMRA, respectively [30]. The intracellular viruses were harvested from the cell pellets after washing twice with PBS to remove extracellular virus. The cell pellets were frozen and thawed for three times to release intracellular DEN-2 virus, then resuspended to 20 μl and subjected to real-time RT-PCR assay.

### Data and statistical analyses

Data are presented as mean ± SEM values. Mann Whiteney U test was used to analyze IL12B and IFNα RNA expression levels between undifferentiated and differentiated THP-1 cells with and without DEN-2 infection. A *P* value of < 0.05 was considered statistically significant. All analyses were performed using SPSS 13.0 (SPSS Inc. Chicago, IL, USA).

## Results

### Undifferentiated THP-1 expressing pDC markers released IFNα but not IL-12 after DEN-2 infection

**Figure 1:**
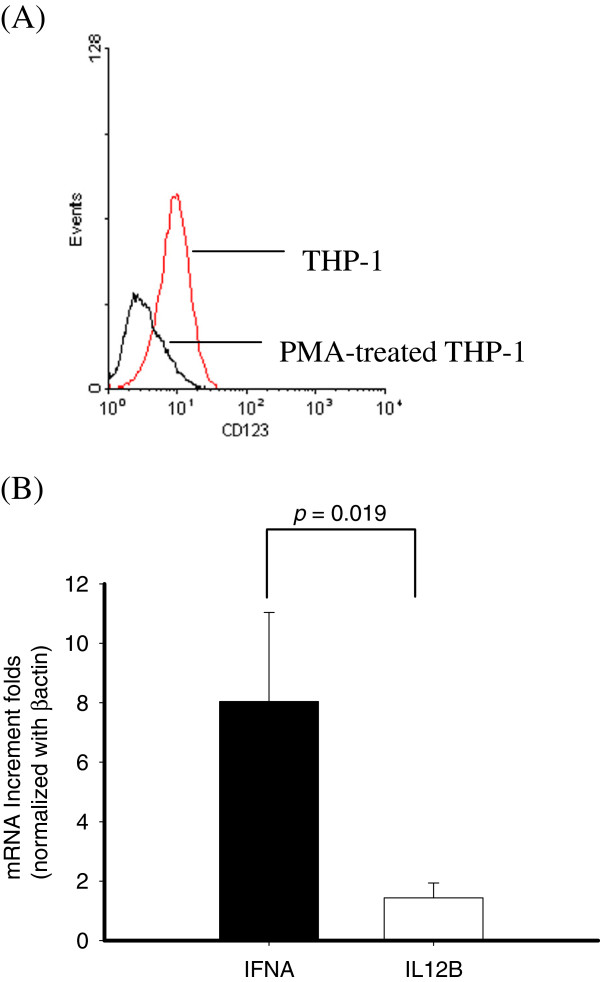
**Flow cytometric analysis of CD123 expression in THP-1 with or without PMA differentiation (A).** Expression of IFNA and IL12B mRNA expression in THP-1 cells with DEN-2 infection **(B)**. Six RNA samples from cells were subjected to testing IFNA (■) and IL12B (□) mRNAs by SYBR Green real-time RT-PCR analysis. (*p* = 0.019).

In order to characterize the expression of cell markers on THP-1 cells, we assessed the CD123 levels, a pDC-specific marker, on THP-1 cells by flow cytometry. As shown in Figure 1A, CD123 was expressed on the surface of THP-1 and down-regulated on the PMA-differentiated THP-1 cells. To investigate whether THP-1 cells elicited Th1 promoting cytokines under DEN-2 infection, cellular RNA was extracted and assessed using real-time RT-PCR after 24 h infection. Higher levels of IFNα mRNA but not IL12B mRNA were induced in post-infected THP-1 cells by DEN-2 (Figure 1B).

### Differentiated THP-1 expressing mDC markers released IL-12 but not IFNα after DEN-2 infection

THP-1 cells grew as a single-cell suspension, and only a few cells spread onto the flask substrate. Approximately 80% of the cells attached to the substrate as early as 3 h after the addition of PMA (8 nM). After a 72-h treatment with PMA, THP-1 spread onto the substrate to be irregular and flattened in shape. Addition of DMSO only at the same dilutions required for obtaining the given concentrations of PMA in culture system did not induce adherence of the cells to the substrate.

**Figure 2:**
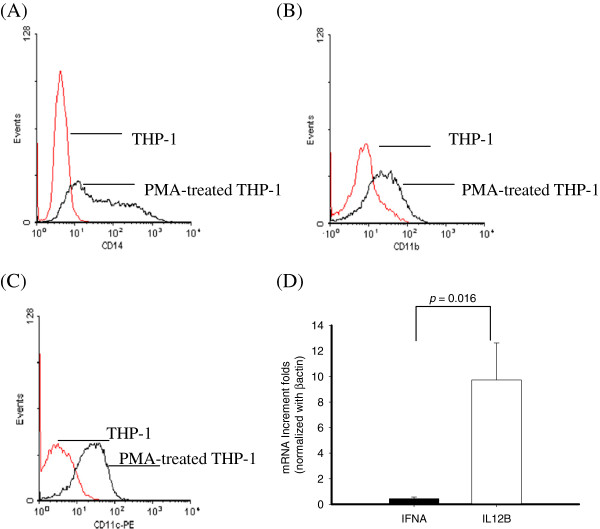
**Flow cytometric analysis of CD14, CD11b, CD11c expression in THP-1 with or without PMA differentiation (A-C).** Expression of IFNA and IL12B mRNA expression in PMA-treated THP-1 cells with DEN-2 infection **(D)**. Six RNA samples from cells were subjected to testing IFNA (■) and IL12B (□) mRNAs by SYBR Green real-time RT-PCR analysis. (*p* = 0.016).

The effect of PMA treatment on the expression of surface markers of THP-1 cells was examined by flow cytometry. THP-1 treated with 8 nM PMA expressed more mDC cell markers such as CD14, CD11b, and CD11c (Figure 2A-C), but not CD123, pDC cell markers (Figure 1A). To analyze whether PMA-treated THP-1 cells elicited Th1 promoting cytokines under DEN-2 infection, cellular RNA was extracted and assessed using real-time RT-PCR after 24 h DEN-2 infection. Higher levels of IL12B mRNA but not IFNα mRNA were induced in PMA-treated THP-1 cells after 24 h DEN-2 infection (Figure 2D).

### Dose dependent and kinetic induction of IFNα /IL12B mRNA in THP-1 cells with or without PMA differentiation after DEN-2 infection

**Figure 3:**
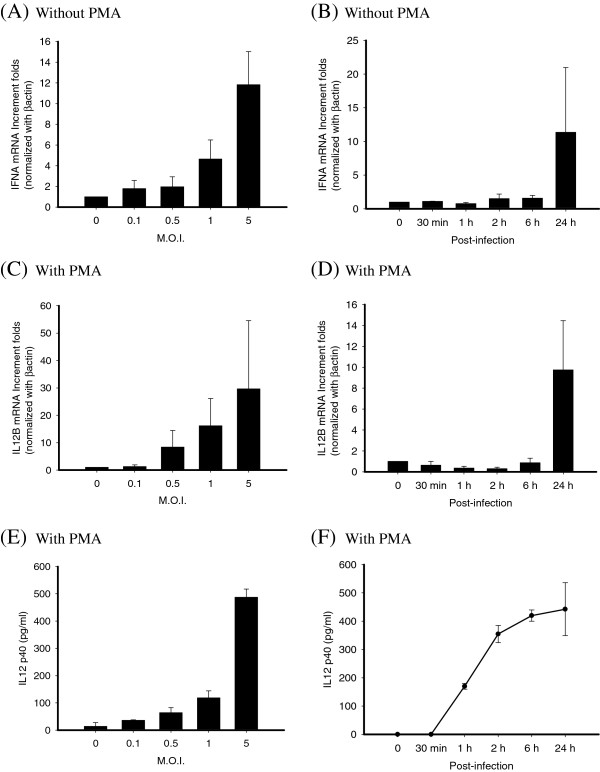
**Dose-dependent and kinetic induction of IFNA and IL12B expression in DEN-2-infected THP-1 cells with or without PMA differentiation.** A series of M.O.I. of DEN-2 were assessed at post-infection 24 h, RNA samples from THP-1 (**A**) or PMA-differentiated THP-1 (**C**) cells were subjected to testing IFNA and IL12B mRNAs by SYBR Green real-time RT-PCR analysis. A different post-infection time of DEN-2 were assessed at M.O.I.=1, RNA samples from THP-1 (**B**) or PMA-differentiated THP-1 (**D**) cells were subjected to testing IFNA and IL12B mRNAs by SYBR Green real-time RT-PCR analysis. The similar dose response **(E)** and kinetic induction **(F)** of IL-12 p40 proteins in the supernatants of DEN-2 infected PMA-differentiated THP-1 cells by ELISA in the same reactions.

Undifferentiated THP-1 cells elicited higher production of IFNα mRNA (Figure 1B); PMA-treated THP-1 cells after DEN-2 infection expressed IL12B mRNA (Figure 2D). To investigate whether Th1 promoting cytokines induced in undifferentiated THP-1 cells were dose dependent, cells were infected by different DEN-2 titers (MOI = 0.1 ~ 5) and RNA were assessed by real-time RT-PCR. As shown in Figure 3A, THP-1 elicited IFNα mRNA levels when cells infected by DEN-2 even at lower MOI, and induced IFNα mRNA reached the maximum at MOI = 5. To analyze whether Th1 promoting cytokines were kinetically induced in THP-1, cells were infected by DEN-2 at MOI = 1 at different time from 30 min, 1 h, 2 h, 6 h, and 24 h. Results showed that the IFNα mRNA expressing levels were kinetically increased in undifferentiated THP-1 cells and reached the maximum at 24 h post-infection of DEN-2 (Figure 3B). Interestingly, the IL12B mRNA expressing levels were also kinetically induced and dose-dependent in PMA-differentiated THP-1 cells and reached its maximum induction at 24 h post-infection of DEN-2 (Figure 3C, D). In order to understand whether the induction of Th1 promoting cytokines were limited to transcriptional level, we assessed the cytokine levels by ELISA in the same reactions. However, we could not detect the IFNα production in the supernatants of DEN-2 infected undifferentiated THP-1 cells. However, only IL-12 p40 proteins were kinetically and dose-dependently detected in PMA differentiated THP-1 cells Results showed that IL-12 p40 production could be induced at 1 h post-infection of DEN-2 and also reached its maximum at 24 h post-infection of DEN-2 in differentiated THP-1 cells, but not in undifferentiated THP-1 cells (Figure 3E, F).

### Down-regulation of IL12B in antibody dependent enhancement of DEN-2 infected differentiated THP-1 cells

**Figure 4:**
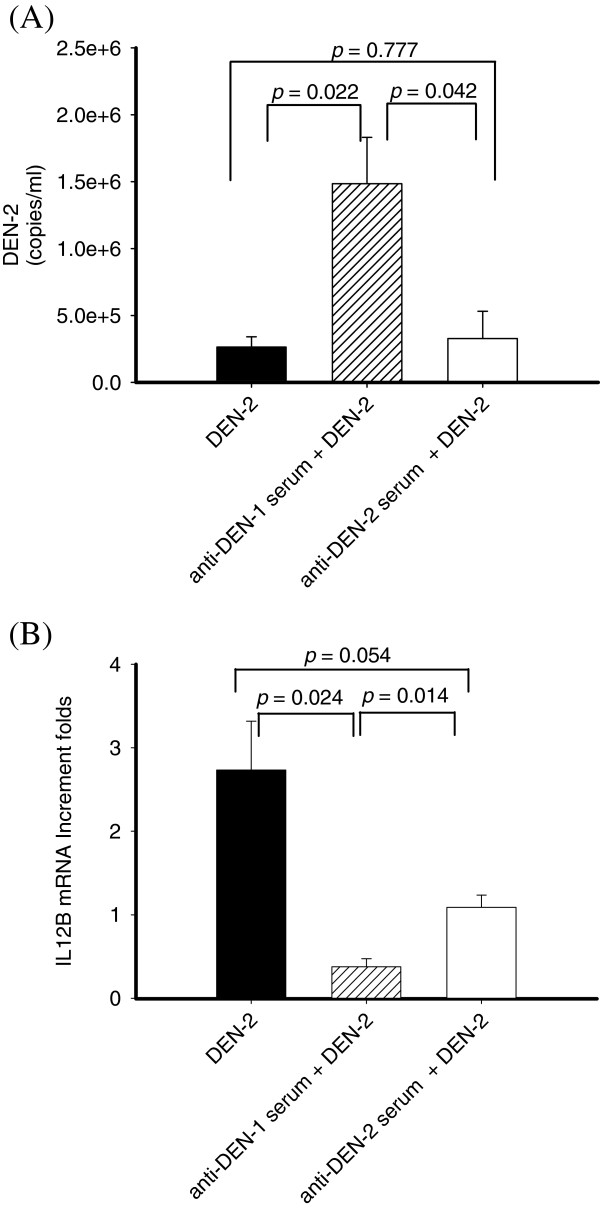
**Enhanced DEN-2 replication but suppressed IL12B in heterotypic antibody-mediated DEN-2 infected differentiated THP-1 cells.** (**A**) To compare viral replication, viral load of DEN-2 in antiserum-mediated DEN-2 infection was determined by TaqMan real-time RT-PCR (DEN-2 (■), anti-DEN1 antiserum + DEN-2 (░), and anti-DEN2 antiserum + DEN-2 (□), *p* = 0.022, 0.042, and 0.777 respectively) after infection of DEN-2 for 24 h in a summary calculated from the results of 5-paired experiments. Suppression of IL12B levels in differentiated THP-1 cells was found in the addition of anti-DEN1 antiserum diluted to 1:1250 after infection of DEN-2 for 24 h (**B**) (DEN-2 (■), anti-DEN1 antiserum + DEN-2 (░), and anti-DEN2 antiserum + DEN-2 (□), *p* = 0.024, 0.014, and 0.054 respectively) after infection of DEN-2 for 24 h in a summary calculated from the results of 5-paired experiments.

Antibody-dependent enhancement of DEN-2 infection occurred when sub-neutralizing antibodies facilitated DEN-2 infection via Fc receptors (FcR). To investigate the role of IL-12 p40 in ADE of DEN-2 infection, we compared the viral replication and IL12B expression in the differentiated THP-1 cell using real-time RT-PCR. As shown in Figure 4A, we found that the sub-neutralizing DEN-1 antiserum could enhance DEN-2 replication in differentiated THP-1 cells from 2.64×10^5^ copies/ml to 1.49×10^6 ^copies/ml in the presence of antiserum at 1:1250 dilution. However, anti-DEN2 antiserum at 1:1250 dilution did not enhance DEN-2 replication in differentiated THP-1 cells (2.64×10^5 ^copies/ml vs. 3.28×10^5^ copies/ml). To measure the expression of IL-12 p40 levels in the ADE of DEN-2 infection of differentiated THP-1 cells, we found that DEN-2 infection induced a higher IL12B expression (Figure 4B). Presence of anti-DEN1 antiserum at 1:1250 dilution, but not anti-DEN2 antiserum, significantly suppressed the DEN-2 induced IL12B expression in differentiated THP-1 cells.

## Discussion

Several prospective studies have concluded that DHF is more common in secondary DEN infections than in primary DEN infections [9,48]. Despite extensive studies, the pathogenesis of DHF cannot be fully attributed to the ADE. Activation of dengue virus-specific T cells and dengue virus-infected monocytes may result in increased capillary permeability in patients with DHF [49,50]. Recently, cytokines related to dominant Th2 reaction have been related to the pathogenesis of DHF [44,48,51,52]. We showed that increase of Th1 cytokine, IL12B expression in differentiated THP-1 cells was found in DEN-2 infection (Figure 3C, D). We also found that heterotypic antibody mediated DEN-2 infection significantly enhanced DEN-2 replication, but suppressed the IL12B expression.

Distinct DC subsets are known to exhibit intrinsic differences in their ability to: 1) regulate the quality of the Th response (Th1, Th2, or cytotoxic T lymphocyte [CTL]); 2) produce antiviral type I IFNs; and 3) cross-present exogenous Ags to CD8^+^ T cells [53]. Our results indicate that treatment of human monocytic leukemia cell line, THP-1, cells with 8 nM PMA for 72 h promotes a differentiation phenotype that is characterized by morphological changes and altered IFNα gene induction. The PMA could induce THP-1 cells to differentiate toward macrophage has been well demonstrated [54-56]. We however demonstrated an up-regulation in expression of mDC-related molecules associated with monocyte differentiation, notably CD11b, CD11c and CD14. Concomitantly, the expression of CD123 was selectively downregulated in the PMA differentiated THP-1 cells. Theses cell surface markers were partly similar to the criteria of differences between myloid DCs and plasmacytoid DCs. THP-1 cells induced elevated IFNα mRNA expression under DEN-2 infection, however, PMA-differentiated THP-1 cells elicited higher IL12B mRNA expression and protein levels under the same infection. The induction of early Th1 cytokines was dose-dependent and time-dependent in THP-1 with or without PMA differentiated cells.

## Conclusion

Human blood DCs can be divided into several distinct phenotypic and functional subpopulations, and both monocytes-derived DCs and PMA-differentiated THP-1 cells are of the myeloid CD11b^+^ CD11c^+^ and CD14^+^, resembling their *in vivo* myeloid counterpart [29]. We showed that THP-1, likes pDCs, responded to DEN-2 by secreting IFNα; on the other hand, PMA-differentiated THP-1 cells, act as mDC, responded to DEN-2 by secreting IL-12. This model may be a good model system for studying early innate immunity of virus infections, and provide a better strategy to prevent infection and complication.

## Abbreviation

ADE: Antibody-dependent enhancement; ALCAM: Activated leukocyte cell adhesion molecule; DC: Dendritic cell; DF: Dengue fever; DHF: Dengue hemorrhagic fever; DMSO: Dimethyl sulfoxide; DSS: Dengue shock syndrome; FBS: Fetal bovine serum; FcγR: Fc-gamma receptors; mDC: Myeloid dendritic cells; pDC: Plasmacytoid dendritic cells; PMA: Phorbol 12-myristate 13-acetate; RT-PCR: Reverse transcriptase-polymerase chain reaction; TLR: Toll-like receptor.

## Competing interests

The authors have declared that no competing interest exists.

## Authors’ contribution

RF carried out the data collection, data interpretation and drafted the manuscript. L carried out the immunoassays and performed the statistical analysis. JT participated in the design of the study. KD conceived of the study, and participated in its design and coordination and helped to draft the manuscript. All authors read and approved the final manuscript.

## Pre-publication history

The pre-publication history for this paper can be accessed here:

http://www.biomedcentral.com/1471-2334/12/340/prepub
